# Empirical estimation of life expectancy from a linked health database of adults who entered care for HIV

**DOI:** 10.1371/journal.pone.0195031

**Published:** 2018-04-05

**Authors:** Dena Schanzer, Tony Antoniou, Jeffrey Kwong, Karen Timmerman, Ping Yan

**Affiliations:** 1 Infectious Disease Prevention and Control Branch, Public Health Agency of Canada, Ottawa, Ontario, Canada; 2 Institute for Clinical Evaluative Sciences, Toronto, Ontario, Canada; 3 Department of Family and Community Medicine, St. Michael's Hospital, Toronto, Ontario, Canada; 4 Li Ka Shing Knowledge Institute, St. Michael's Hospital, Toronto, Ontario, Canada; 5 Department of Family & Community Medicine, University of Toronto, Toronto, Ontario, Canada; 6 Public Health Ontario, Toronto, Ontario, Canada; 7 Dalla Lana School of Public Health, University of Toronto, Toronto, Ontario, Canada; 8 University Health Network, Toronto, Ontario, Canada; Giuseppe Vittorio De Socio, Azienda Ospedaliera Universitaria di Perugia, ITALY

## Abstract

**Background:**

While combination antiretroviral therapy (cART) has significantly improved survival times for persons diagnosed with HIV, estimation of life expectancy (LE) for this cohort remains a challenge, as mortality rates are a function of both time since diagnosis and age, and mortality rates for the oldest age groups may not be available.

**Methods:**

A validated case-finding algorithm for HIV was used to update the cohort of HIV-positive adults who had entered care in Ontario, Canada as of 2012. The Chiang II abridged life table algorithm was modified to use mortality rates stratified by time since entering the cohort and to include various methods for extrapolation of the excess HIV mortality rates to older age groups.

**Results:**

As of 2012, there were approximately 15,000 adults in care for HIV in Ontario. The crude all-cause mortality rate declined from 2.6% (95%CI 2.3, 2.9) per year in 2000 to 1.3% (1.2, 1.5) in 2012. Mortality rates were elevated for the first year of care compared to subsequent years (rate ratio of 2.6 (95% CI 2.3, 3.1)). LE for a 20-year old living in Ontario was 62 years (expected age at death is 82), while LE for a 20-year old with HIV was estimated to be reduced to 47 years, for a loss of 15 years of life. Ignoring the higher mortality rates among new cases introduced a modest bias of 1.5 additional years of life lost. In comparison, using 55+ as the open-ended age group was a major source of bias, adding 11 years to the calculated LE.

**Conclusions:**

Use of age limits less than the expected age at death for the open-ended age group significantly overstates the estimated LE and is not recommended. The Chiang II method easily accommodated input of stratified mortality rates and extrapolation of excess mortality rates.

## Introduction

Since the introduction of combination antiretroviral therapy (cART) for the treatment of HIV in 1996, survival has improved considerably from an estimated 11 months for persons diagnosed with an AIDS-defining illness in 1984 [1] to an estimated life expectancy (LE) that approaches that of the general population in the post-cART period [2]. The number of deaths due to HIV in Canada has declined from 1764 in 1995 to 276 in 2012 [3], and all-cause mortality rates among HIV-positive persons have declined considerably during the post-cART period [4].

LE is a statistical estimate of the average number of years of life remaining as of a specific age for a specific group of individuals. It is widely used to assess the health of the population of one country in comparison with other countries, over time, or across socio-demographic segments of the population. As future mortality rates are unknown, the calculation, known as the abridged life table approach or period life table approach, is based on mortality rates for 5-year age groups for a specific period. Two important issues arise with the calculation of LE for disease-specific cohorts. One issue is that mortality rates become a function of both age and time since disease onset (or more generally, time since entering the cohort). For HIV, as many patients are diagnosed with late-stage disease, the risk of mortality is expected to be higher shortly after diagnosis than in patients of the same age (and over the same period of time) who were diagnosed earlier and have already responded to treatment. Secondly, as a disease with onset primarily in young adults, estimates of mortality rates for the oldest 5-year age groups may not be available or may be too imprecise due to the paucity of cases in the oldest age groups, especially if one stratifies by time since entry into the cohort.

Despite these limitations, LE and its counterpart, years of life lost (YLL), are commonly used summary measures of disease burden and are important inputs for cost-effectiveness analyses of public health interventions. The earliest estimates of LE used the Kaplan-Meier survival estimator, as mortality rates were primarily a function of time since diagnosis rather than age. As survival rates improved, however, LE estimation has become more complex with the need to account for the impact of age on mortality rates. Once the median survival time exceeded the follow-up time, relative survival methods based on proportional hazards models were used to extrapolate mortality rates to follow-up times and ages beyond the study data. However, proportional hazards models assume that the ratio of the age-specific mortality rates for patients with HIV compared to the general population is constant. Rather, the ratio was found to decline with increasing age [5,6], leading to the acknowledgment that extrapolation to older age groups using a constant rate ratio, as required in a proportional hazards model, is questionable.

A recent review article [7] found that most Canadian estimates of LE were based on an abridged life table approach. This approach uses 5-year age-specific mortality rates to calculate LE based on current period mortality rates, though the oldest age group is always open. As a rule of thumb, the oldest 5-year age group should be older than the anticipated average age at death. However, a threshold of 5000 for the person-years at risk for each age group is also recommended [8]. As the number of persons in the HIV cohort for the older 5-year age groups was considered too small to estimate mortality rates for the older age groups, most studies from developed countries using an abridged life table approach combined the older age groups and used a 55+ age group as the open-ended age group [7]. Improved survival rates, however, put the use of a 55+ open-ended age group in violation of the first rule of thumb. Most studies also ignored the differences in mortality rates among recent entries to the cohort compared to longer-term survivors. Hence, there are concerns about the potential for bias in many of these studies. These issues are common to most restricted cohorts with a selected entry, such as for life insurance applicants or breast cancer survivors. The recommended approach is to calculate mortality rates as a function of both age and time elapsed since entry, using some form of mathematical smoothing with extrapolation to the older age groups [9]. When the select effect is not negligible and the population at risk is small, as is the case with our HIV cohort, a modified abridged life table approach can still be used, though additional effort is required to develop appropriate mathematical formulae for mortality risk as a function of age and time since selection that incorporate smoothing or extrapolation to older age groups. [9].

It has also been noted that time since starting cART is less important for predicting mortality risks in HIV patients who have survived the initial period of treatment [10,11]. However, using pooled mortality risks (i.e., pooling the higher mortality risks observed during the first couple of years following diagnosis with the lower mortality risks of persons of the same age who have already responded to treatment) has a potential for bias. One study documented that the standard abridged life table estimate of LE (using pooled mortality estimates) was approximately one-half the estimate from the relative survival model (using the proportional hazards assumption) [11]. This difference was attributed in part to the inclusion of deaths from new diagnoses in all age-specific mortality rates. That is, the mortality rate of a case newly diagnosed at age 50 would likely be much higher than the rate at age 50 for someone diagnosed at age 20. In Wanderler’s review study, Canadian estimates of LE at age 20 for persons with HIV varied from 31 to 51 years [7]. Although LE varies considerably with established risk factors such as viral load and CD4 count at the start of treatment and lifestyle behaviours such as injection drug use [2,10,12], methodological differences quite possibly contributed to much of the variation across studies as most of the HIV cohorts in the review study were not for specialized sub-populations.

In this study, we aimed to: 1) modify the standard abridged life table calculator to accept age-specific mortality rates stratified by time since entering the cohort, and to use projected mortality rates; 2) estimate LE for a Canadian population-based cohort of persons in care for HIV and put this in the context of other trends, such as incidence, prevalence and mortality rates for this cohort; 3) evaluate the impact of methodological differences on the LE estimates; and 4) provide access to the modified LE calculator for ease of use with other cohorts.

## Methods

### Sources of data

The Institute for Clinical Evaluative Sciences (ICES) updated an existing cohort of persons aged 18 years of age or older and in care for the treatment of HIV in Ontario, Canada. The linked data set was created by applying a validated case-finding algorithm for HIV to ICES health administrative data holdings, including health insurance registration records, physician billing claims, and hospitalization and mortality records [13]. The available data covers a period from 1991 to 2015. However, the case-finding algorithm requires three HIV-related visits within a three-year period, thereby limiting incidence, prevalence and mortality estimates to patients entering care or in care, respectively, between 1994 and 2012. The first of three HIV consultation dates was considered the date of physician diagnosis (entering medical care for HIV). Ontario residents are eligible to register with the Ontario Health Insurance Plan (OHIP) and receive a unique provincial health card number. It is this number that is used to link the ICES data holdings used in this study. Date of death was obtained by linkage of the HIV cohort with the death database. Date of death is complete as of December 31, 2014 as long as the case remains an Ontario resident. To calculate person-time at risk of death (registered with an OHIP number), we used a combination of the date of the end of OHIP eligibility and date of last health care contact to get an approximate estimate of the date the case moved out of the province (for cases who had no health care contact for at least 3 years prior to March 31, 2015). Unfortunately, the former appeared to be incomplete. Hence, the end of follow-up for cases without a date of death was set as the minimum of the date of end of eligibility and one year after the date of last health care contact. The resulting loss to follow-up among the HIV cohort was approximately 1% per year, and was concentrated among younger age groups as reflected in the interprovincial migration data (Ontario residents migrate out of the province at a rate of approximately 2% per year [14]). This rule added on average a half year to the end date per person deemed lost to follow-up. The linked database included the following potential predictors of mortality: sex, neighbourhood income quintile, and urban/rural residency. Date of birth for the calculation of age and annual age-specific mortality rates is available within the ICES environment, but was not available via the remote access system through which we accessed the data. The remote access database included 5-year birth cohorts with all cases born before 1940 grouped into one open-ended birth cohort by ICES, in order to comply with additional privacy restrictions placed on potentially identifying information such as year of birth. This restriction applied to the remote access database only. An additional table of mortality rates by 5-year age groups for the 2005–2012 period was provided by ICES staff directly from the full ICES database in order to assess the impact of this restriction. Measures of disease severity at the time of diagnosis were not available from the remote access database. Mortality rates by 5-year age groups for the population of Ontario were obtained from Statistics Canada [15].

#### Data access

All data processing was done on site at ICES either by ICES staff or remotely through their IDAVE system by the study PI. Access via a remote connection was limited to anonymized de-identified records with restrictive group identifiers related to year of birth. The study data remains the property of ICES and REB approval was required to access ICES data holdings remotely. Interested researchers may contact Data and Analytic Services at ICES for more information on how to access their data holdings at www.ices.on.ca/DAS or via e-mail at das@ices.on.ca [16]. Minimal datasets have been provided where feasible as supplementary files.

### Statistical analysis

Logistic regression was used to assess risk factors for mortality rates and determine the appropriate level of stratification for time since entry. Annual trends were estimated using quasi-Poisson regression over the post cART period from 2000 to 2012.

To calculate LE at age 20 years for the reference population, we used the standard abridged life table with 5-year age groups for ages 20–89 years with an open-ended age group of 90+. For the HIV cohort, we calculated mortality rates over a period from 2005 to 2012 for 5-year age groups (where HIV case counts permitted), stratified by time since diagnosis (1^st^ year of care, and 1^st^ year survivors, as indicated by the results of the logistic regression). Stratification was achieved in the LE calculator by dividing the first 5-year age group into two age groups (of 1-year and 4-year durations, see S1 Zip File). The 2005–2012 period was chosen to meet ICES [16] cell size limitations for publication of tables. As for most estimates of LE, we used a period LE approach, where life expectancy is defined as the expected number of years of life remaining at a given age, assuming that the age-specific mortality rates of the most recent period remain unchanged. For methodological comparisons, we primarily used LE at age 20. Noting that few cases enter care before age 20, we also calculated the average LE based on the age distribution of persons entering care.

The main analysis focused on using the remote access database, where age-specific mortality rates were not available after age 60 (since persons born before 1940 could not be placed in an appropriate 5-year age group). As mortality rate estimates are required for all age groups to calculate LE, we used various extrapolation methods to obtain estimates for the age groups over the age 60 (60–64, 65–69, 70–74, 75–79, 80–84, 85–89, and 90+). This dataset likely represents more closely the information available to previous studies that chose to use the 55+ age cohort as the open-ended age group. Even with data from the specially prepared table of mortality rates by 5-year age groups (see Tab E in S1 Tables), we used the same extrapolation procedure to adjust the HIV mortality rate for the 90+ age group because of the small number of HIV cases in this age group and out of concern for potential differences in the age distribution within the 90+ age group. Unless otherwise specified, summary LE estimates are based on the stratified age-specific mortality rates from the full ICES database (see Tab E in S1 Tables).

With small populations in the older age groups of the HIV cohort, the Chiang II modification for the abridged life table method is recommended [8,17–19], as this modification can handle age groups with zero deaths, and reference mortality rates can be substituted. Three options recommended for the extrapolation of mortality rates and calculation of LE in cohorts with chronic medical conditions [20] were evaluated in a sensitivity analysis: 1) Gompertz’s law (which assumes that the force of mortality increases exponentially with age [20]); 2) extrapolation based on a constant relative mortality rate or rate ratio; and 3) extrapolation based on a constant excess mortality rate or rate difference, compared to the general population. In addition, we also compared variations in the oldest age group (extrapolation starting at age 60 and age 90, as well as use of an open-ended 55+ age group). The 55+ open-ended age group option has the advantage that the Chiang II modification is not required to calculate LE. However, the main concern with using a 55+ open-ended age group is that the age distributions within this large age group are different (resulting in a lower mortality rate for the HIV cohort than for the general population).

A SAS [21] macro was written to calculate LE using the Chiang II method [8,17,18], with modifications to accept mortality rates stratified by time since entering the cohort as input and to include the three extrapolation options (S1 Zip File). The macro also requires mortality rates for the reference population as input. In this way, the projection algorithm ensures that the extrapolated mortality rates in the oldest age groups are not lower than those for the general population. The analyses were performed with SAS (version 9.3; Cary, NC) [16,21].

## Results

**Fig 1:**
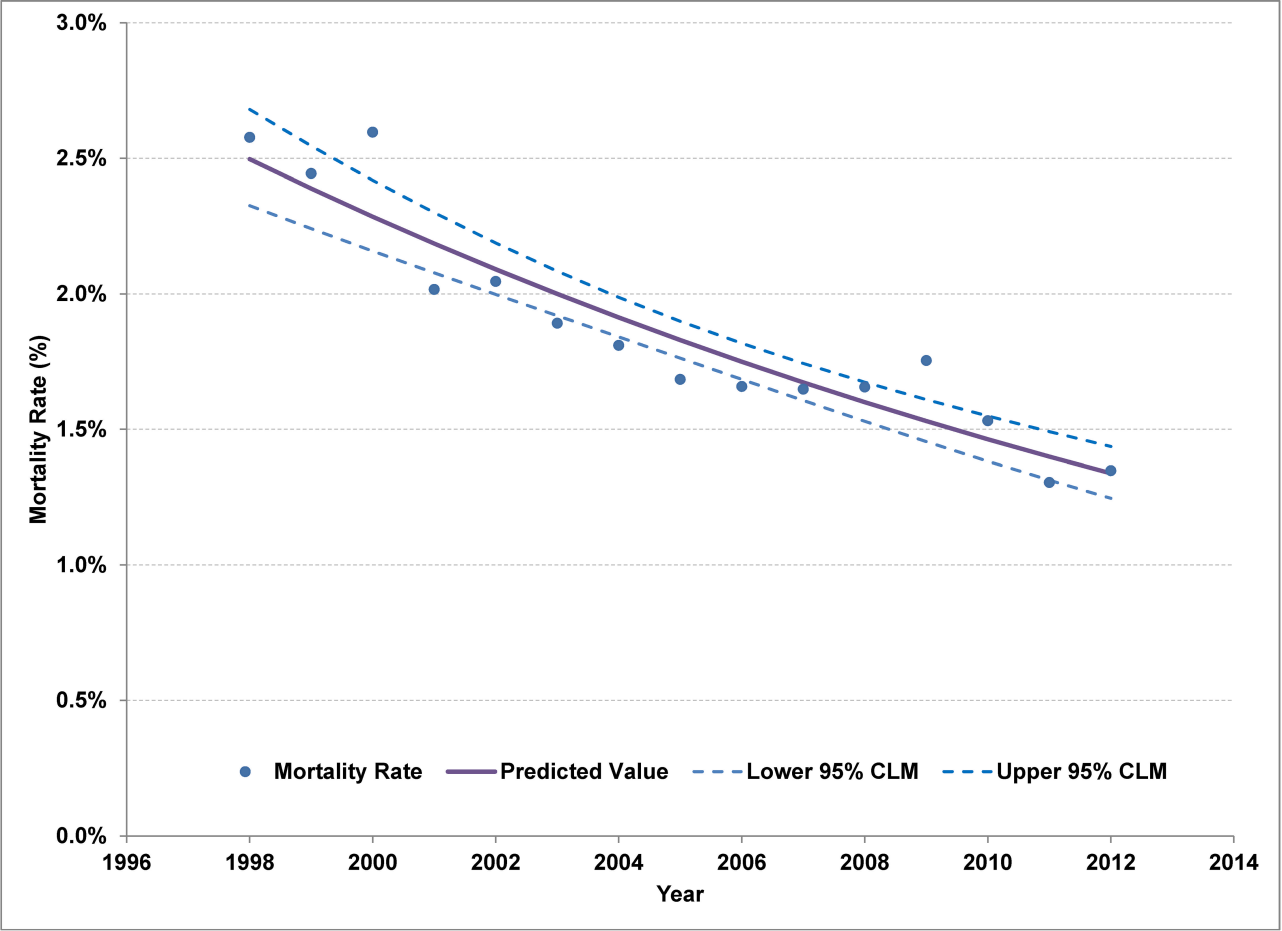
Trend in crude all-cause mortality rate for persons in care for HIV, Ontario 1994–2012.

**Fig 2:**
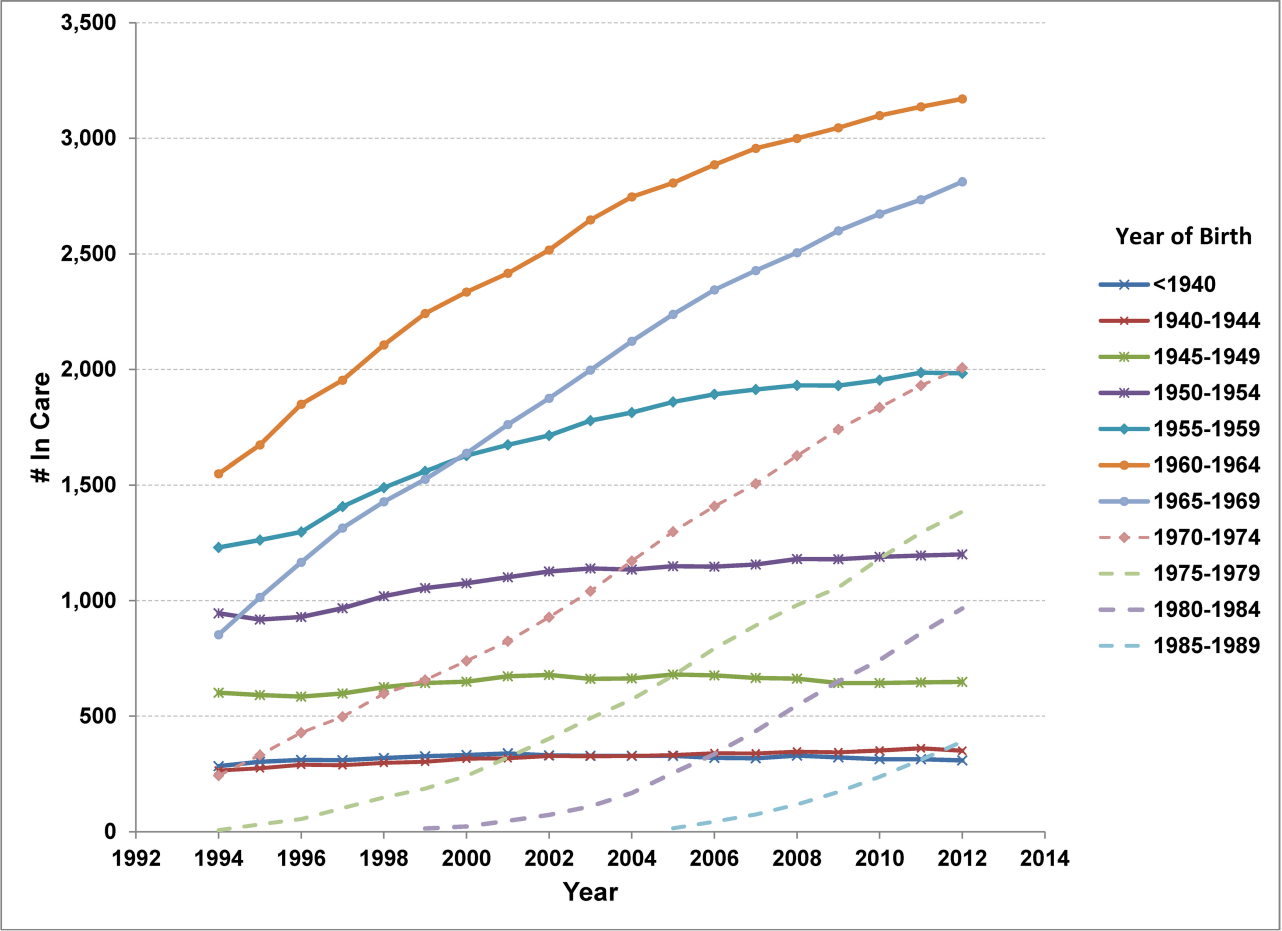
Number in care for HIV by 5-year birth cohort and year, Ontario, 1994–2012.

**Fig 3:**
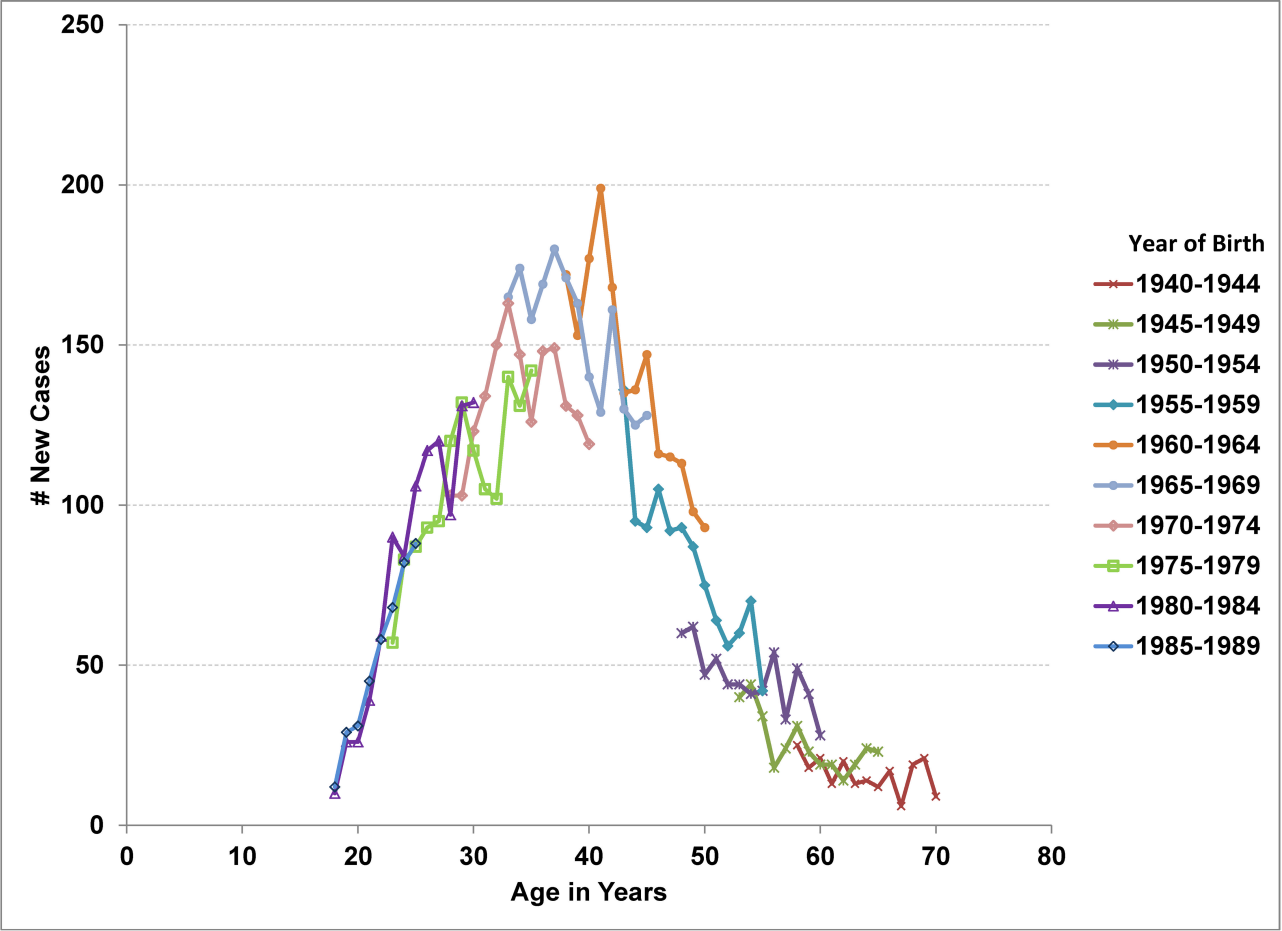
Number entering care for HIV by birth cohort and median age of the cohort, Ontario, 2000 to 2012. Each line corresponds to the number entering care in 2000 (left most point) to 2012 (right most point) for one five-year birth cohort. With each year, the birth cohort ages 1 year. With age as the x-axis, the increase in the number of new cases between the ages of 45 and 55 years in recent years is notable. The increase corresponds to the aging of the high prevalence birth cohorts (1960–64 and 1965–69).

As of the end of 2012, there were approximately 15,000 adults alive and in care for HIV in Ontario (Table 1). The all-cause crude mortality rate had declined from 2.6% (95%CI 2.3, 2.9) in 2000 to 1.3% (95%CI 1.2, 1.5) in 2012 for an average annual percentage change (AAPC) of -4.2% (95%CI -5.3, -3.1) (Fig 1). From 2000 to 2012, there were approximately 850 new adult cases entering care in Ontario per year, 200 deaths (all causes) and 120 lost to follow-up (moved out of province). As a result, the prevalence of adults in care for HIV increased 3.9% (95%CI 3.8, 4.0) annually. Though the number of persons entering care for HIV has been stable in recent years, a 12% decline in new cases was observed for persons younger than 45 years of age between 2003–08 and 2009–2012 (AAPC of -2.3% (95%CI -3.1%, -1.6%)) (Table 2). This decline was offset by a significant increase of 6.3% per year (95%CI 4.1, 8.5) in new cases among older adults. Persons born in the 1960s had the highest HIV prevalence in care as of 2012 (Fig 2, Tab B in S1 Tables) as well as the highest incidence of entering care over the 2000 to 2012 period (Fig 3, Tab C in S1 Tables). Though the number of cases entering care had started to drop for the 1960–64 and 1965–69 birth cohorts once these cohorts reached an age of 40 to 45 years, incidence for the 1960s birth cohorts was higher than for the 1950s birth cohorts at similar ages (Tab C in S1 Tables, Fig 3). The declining trend from age 40 to 60 years was similar for each 5-year birth cohort for which we have data at that age, with an average decline of 6.2% (95% CI 5.0, 7.2) per year of age. The shift towards older ages at entry to care is apparent in the figure in Tab C of S1 Tables.

**Table 1:** Patients in care for HIV in Ontario: Incidence, prevalence and all-cause deaths, 1994 to 2012.

Year	New Cases	Deaths	LTF ^a^	In Care	% LTF	% New Cases	Crude Mortality Rate	95% CI
**1994**	1259	628	97	5976	1.6%	21.1%	10.5%	(9.7%, 11.3%)
**1995**	1198	667	107	6400	1.7%	18.7%	10.4%	(9.6%, 11.2%)
**1996**	1121	508	104	6909	1.5%	16.2%	7.4%	(6.7%, 8.0%)
**1997**	935	312	96	7436	1.3%	12.6%	4.2%	(3.7%, 4.7%)
**1998**	905	207	101	8033	1.3%	11.3%	2.6%	(2.2%, 2.9%)
**1999**	777	208	91	8511	1.1%	9.1%	2.4%	(2.1%, 2.8%)
**2000**	801	233	104	8975	1.2%	8.9%	2.6%	(2.3%, 2.9%)
**2001**	784	191	95	9473	1.0%	8.3%	2.0%	(1.7%, 2.3%)
**2002**	788	204	85	9972	0.9%	7.9%	2.0%	(1.8%, 2.3%)
**2003**	847	199	100	10520	1.0%	8.1%	1.9%	(1.6%, 2.2%)
**2004**	857	200	126	11051	1.1%	7.8%	1.8%	(1.6%, 2.1%)
**2005**	892	196	110	11637	0.9%	7.7%	1.7%	(1.4%, 1.9%)
**2006**	870	202	120	12185	1.0%	7.1%	1.7%	(1.4%, 1.9%)
**2007**	841	209	134	12683	1.1%	6.6%	1.6%	(1.4%, 1.9%)
**2008**	861	219	101	13224	0.8%	6.5%	1.7%	(1.4%, 1.9%)
**2009**	841	240	139	13686	1.0%	6.1%	1.8%	(1.5%, 2.0%)
**2010**	881	218	113	14236	0.8%	6.2%	1.5%	(1.3%, 1.7%)
**2011**	901	193	145	14799	1.0%	6.1%	1.3%	(1.1%, 1.5%)
**2012**	858	206	169	15282	1.1%	5.6%	1.3%	(1.2%, 1.5%)

^a^ Lost to follow-up

**Table 2:** Change in distribution of age at diagnosis from 2003 to 2012.

	Year of Diagnosis					
Age Group	2003–2007	(%)	2008–2012	(%)	% Change ^a^	AAPC (95%CI) ^b^
**<45**	3214	75%	2825	65%	-12%	-2.3% (-3.1%, -1.6%)
**45+**	1093	25%	1514	35%	39%	6.3% (4.1%, 8.5%)
**Total**	4307		4339		1%	0.2% (-0.4%, 0.7%)

^a^ % change between 5-year periods

^b^ AAPC: Average Annual Percentage Change calculated with trend estimated using quasi-Poisson regression with log link.

**Fig 4:**
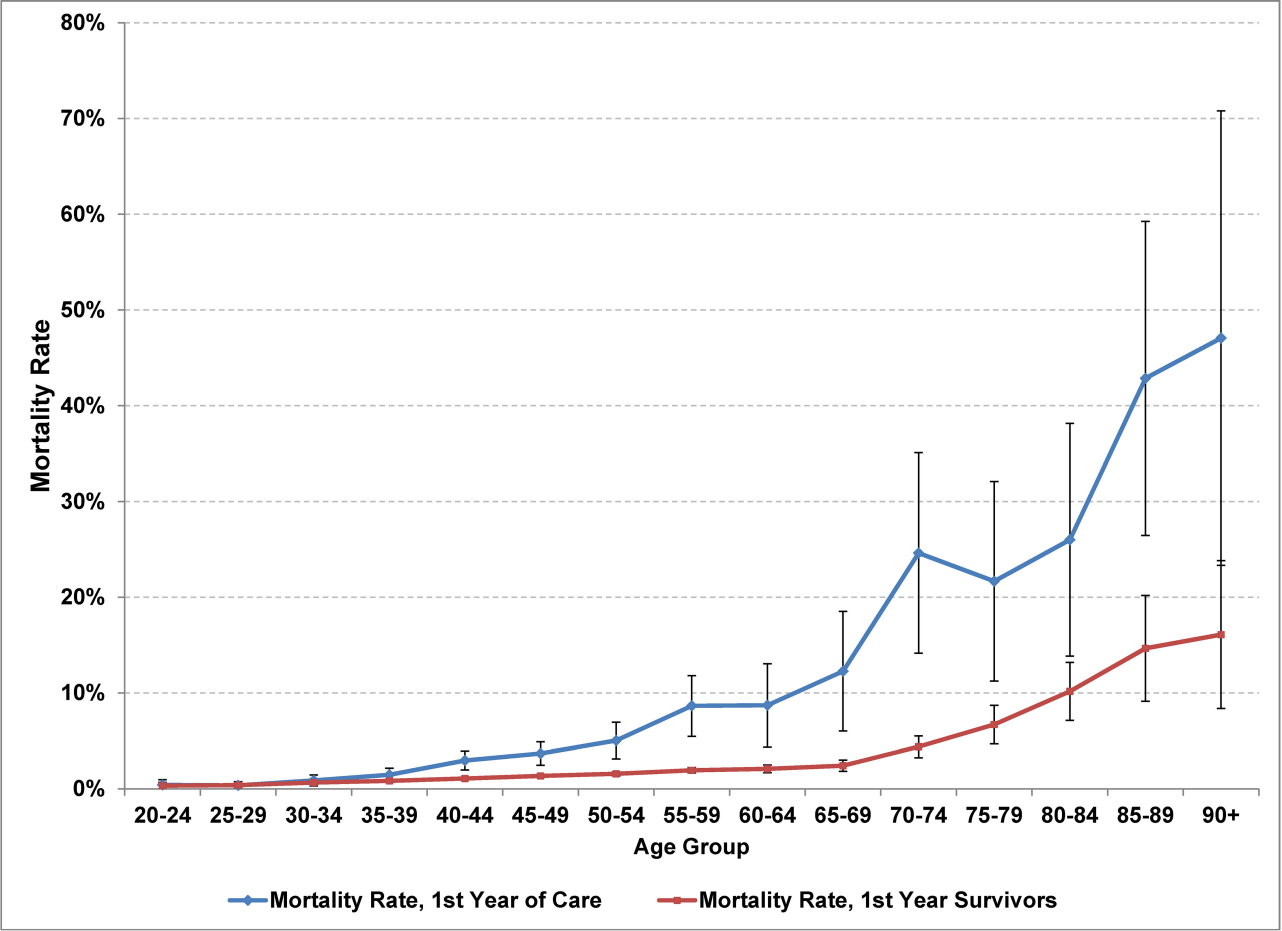
All-cause mortality rates among persons in care for HIV by 5-year age group and time since entering care, Ontario, 2005–2012. Mortality rates are shown with 95%CI for 1^st^ year of care and for 1^st^ year survivors.

**Fig 5:**
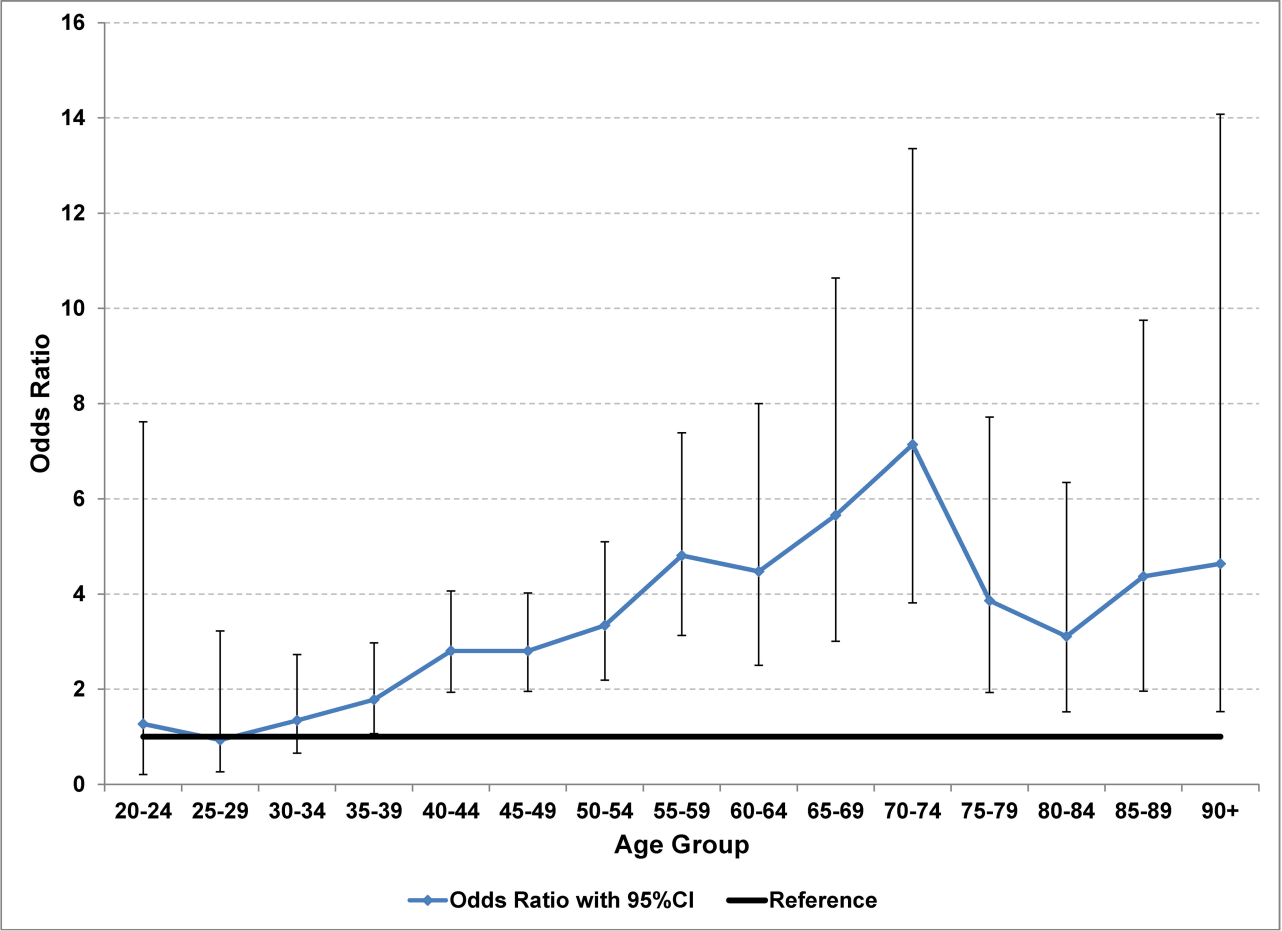
Odds of dying during the 1^st^ year of care for HIV compared to 1^st^ year survivors, by age group, Ontario, 2005–2012.

Mortality rates were elevated in the first year of care compared to mortality rates in subsequent years (OR = 3.1; 95% CI 2.7, 3.6). The OR for more than 2 years in care compared to the second year in care was not statistically significant at 0.85 (95%CI 0.68, 1.07), nor was sex a statistically significant predictor of mortality rates for the HIV cohort. Mortality rates were slightly elevated in the poorest income quintile (OR 1.2, 95%CI 1.1, 1.4) and lower in the richest income quintile (OR 0.84, 95%CI 0.72, 0.98) compared to the middle 3 income quintiles (Tab D in S1 Tables). The risk of dying during the first year of care was elevated compared to subsequent years starting with the 35–39 age group (OR 1.8, 95%CI 1.1, 3.0) and increased with increasing age, possibly peaking with the 70–74 age group (OR 7.1, 95%CI 3.8, 13.4) (Table 3, Fig 4, Fig 5, Tab E in S1 Tables).

**Table 3:** All-cause mortality rates among persons in care for HIV by time since entering care and 5-year age group, with rate ratios and odds of dying during first year of care compared to subsequent years, and rate ratios and rate differences for the HIV cohort compared to the general population, Ontario, 2005–2012.

Age Group	Mortality Rate, 1st year of care	Mortality Rate, 1st year survivors	OR	95%CI	RR	95%CI	Ontario Mortality Rate (reference)	Rate Difference ^a^	Rate Ratio ^a^
20–24	0.004	0.0031	1.3	(0.2, 7.6)	1.3	(0.2, 7.6)	0.00045	0.0027	7.0
25–29	0.003	0.0037	0.9	(0.3, 3.2)	0.9	(0.3, 3.2)	0.00046	0.0033	8.1
30–34	0.009	0.0065	1.3	(0.7, 2.7)	1.3	(0.7, 2.7)	0.00055	0.0060	11.8
35–39	0.015	0.0083	1.8	(1.1, 3.0)	1.8	(1.1, 2.9)	0.00075	0.0075	11.0
40–44	0.030	0.0107	2.8	(1.9, 4.1)	2.7	(1.9, 3.9)	0.00119	0.0095	9.0
45–49	0.037	0.0135	2.8	(2.0, 4.0)	2.7	(1.9, 3.9)	0.00192	0.0115	7.0
50–54	0.050	0.0156	3.3	(2.2, 5.1)	3.2	(2.2, 4.8)	0.00317	0.0124	4.9
55–59	0.086	0.0193	4.8	(3.1, 7.4)	4.5	(3.0, 6.7)	0.00492	0.0144	3.9
60–64	0.087	0.0209	4.5	(2.5, 8.0)	4.2	(2.4, 7.1)	0.00758	0.0133	2.8
65–69	0.123	0.0241	5.7	(3.0, 10.6)	5.1	(2.9, 8.9)	0.01198	0.0121	2.0
70–74	0.246	0.0438	7.1	(3.8, 13.4)	5.6	(3.4, 9.3)	0.01926	0.0245	2.3
75–79	0.217	0.0669	3.9	(1.9, 7.7)	3.2	(1.8, 5.7)	0.03213	0.0348	2.1
80–84	0.260	0.1016	3.1	(1.5, 6.3)	2.6	(1.5, 4.5)	0.05555	0.0460	1.8
85–89	0.429	0.1465	4.4	(2.0, 9.7)	2.9	(1.7, 5.0)	0.09613	0.0504	1.5
90+^b^	0.471	0.1609	4.6	(1.5, 14.1)	2.9	(1.5, 5.9)	0.20184		

^a^ Compared to the reference population.

^b^ The age distribution within the open-ended age group (90+) is expected to be considerably younger for the HIV cohort compared to the general population, hence the lower mortality rate for the HIV cohort.

Compared to the general population of Ontario, the age-specific mortality rate differences increased with increasing age, while the rate ratio decreased with increasing age (Table 3). Mortality rates by 5-year age groups, including the population at risk and number of deaths stratified by time since entering care, are provided in Tab E in S1 Tables for the 2005–2015 period (input to run the LE calculations). As expected with an emerging disease, the precision of the mortality rate estimates for older age groups becomes increasingly poorer. Note that trends in the excess mortality rate by age observed from age 20 to 59 were not a good predictor of excess mortality observed at older ages and that declines in the rate ratios observed over the same ages did not continue to older ages (see figure in Tab E in S1 Tables).

**Fig 6:**
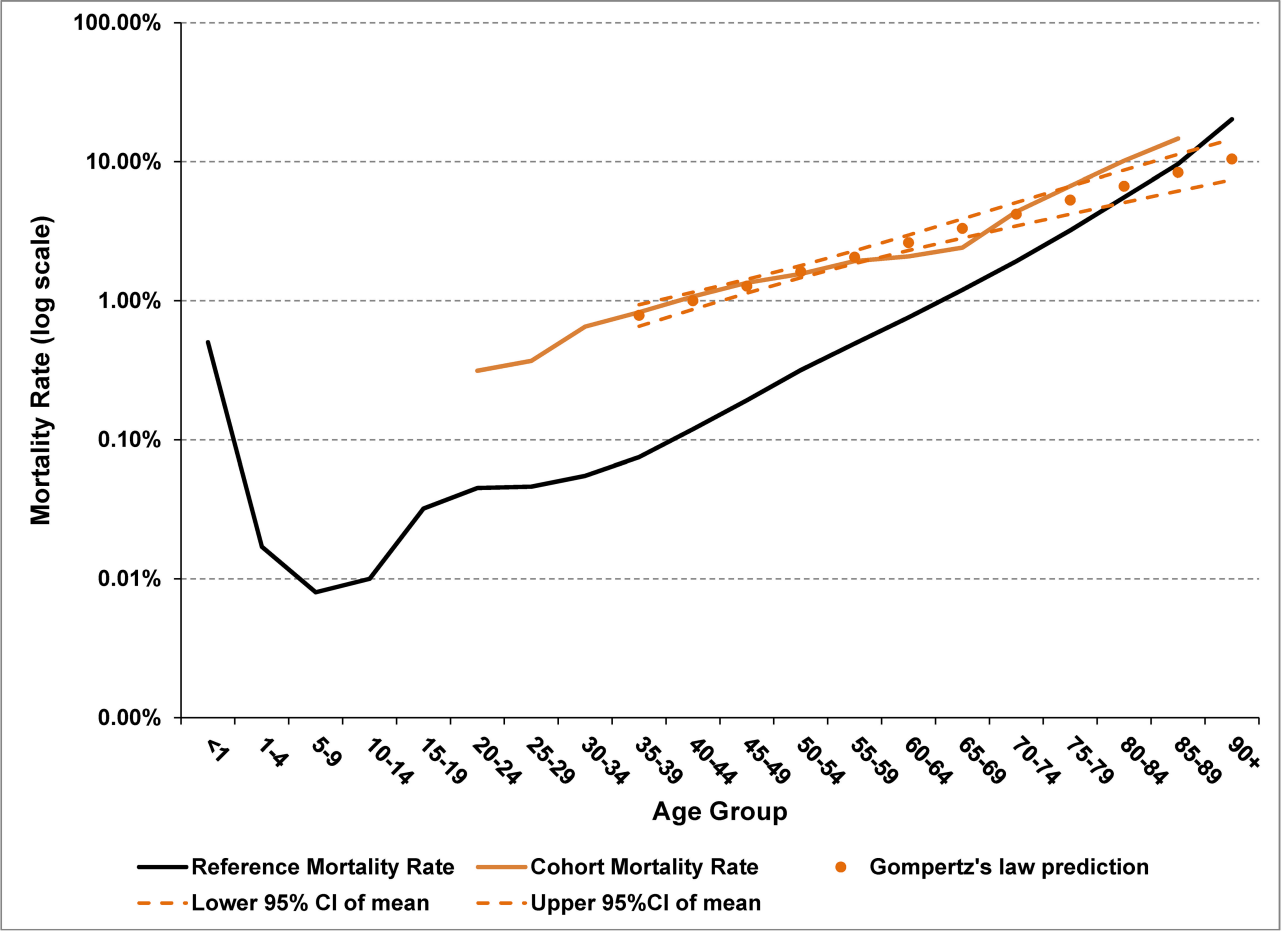
Mortality rates by 5-year age group for HIV cohort with extrapolation using Gompertz’s law, log scale. Gompertz’s law of exponential increase in mortality rates with age is a good fit to the data corresponding to ages 30 to 59 years, as noted by the tight 95%CI for the mean predicted value. Note that the Gompertz’s projection for HIV mortality rates does not sufficiently account for the effects of aging, as it crosses the mortality curve for the general population.

The Gompertz projection of HIV mortality rates with extrapolation starting at age 90 gives a LE of 47.5 years for a 20-year old entering care for HIV, compared to 62.2 years for the reference population, which corresponds to an estimated YLL of 14.7 years. Similar results were obtained using a rate ratio of 2.0 for extrapolation (LE of 47.3 years) or Gompertz projection starting at age 60 (LE of 48.3) (Table 4). The age-specific mortality rates for persons living with HIV were reasonably characterized by Gompertz’s law of exponential growth (Fig 6, Tab F in S1 Tables), though the Gompertz projection had a tendency to underestimate mortality rates for the oldest age groups. (Note that the trend lines cross at approximately age 80 in Fig 6) A projected rate ratio of 3 starting at age 60 reduced the calculated LE by an additional 3 years (Table 4). However, using 55+ as the open-ended age group resulted in a LE of 58.6 years (compared to 47.5), whereas ignoring the higher mortality rates during the first year of care (pooled mortality rates) resulted in a reduction of 1.5 years in LE. The precision of the LE estimates are represented in Table 4 by the error bar corresponding to the 95% CI, which is approximately +/- 1 year for most LE calculations for the HIV cohort at age 20. The estimate of the precision corresponds to the uncertainty associated with the years for which age-specific mortality rates are available. Much of this uncertainty comes from the younger age groups due to the small number of cases (see Tab F in S1 Tables) and the resulting large coefficient of variation for the age-specific mortality rate (see Tab E in S1 Tables). This sensitivity analysis suggests that the additional uncertainty from unknown mortality rates for older ages is much less than the bias introduced with using 55+ as the open-ended age group.

**Table 4:** Sensitivity analysis for life expectancy extrapolation options using Chiang II abridged lifetable for male and female combined, 2005–2012.

		Extrapolation Method	Age starting extrapolation	LE	95%CI Error bar	YLL
**At 20 years of Age**						
		Reference Population	none	62.2	0.03	
	**Mortality Rates for 5-year age groups 20–59 yrs**					
		Gompertz	60	48.3	0.95	14.0
		Rate Ratio of 2.0	60	47.4	0.93	14.8
		Rate Ratio 3.0	60	45.5	0.88	16.8
		Rate Difference of 0.013	60	48.5	0.96	13.7
		Rate Difference of 0.02	60	47.3	0.93	14.9
		Rate Difference of 0.03	60	45.9	0.89	16.3
	**Not stratified by time since entering care**					
		Rate Difference of 0.017 ^a^	60	47.2	0.92	15.0
		Gompertz ^a^	60	46.8	0.91	15.5
	**Includes actual Mortality Rates for 60–89**					
		Gompertz	90	47.5	1.12	14.7
		Rate Ratio of 2.0	90	47.3	1.09	14.9
	**Without extrapolation**					
		55+ open-ended Age Group	none	58.6	1.18	3.7
**At Age 55 years of age (and surviving 1st year of care)**						
		Reference Population	none	28.9	0.02	
	**Mortality Rates for 5-year age groups 20–59 yrs**					
		Gompertz	60	23.1	0.30	5.8
		Rate Ratio of 2.0	60	22.0	0.28	6.9
		Rate Ratio 3.0	60	19.3	0.24	9.6
		Rate Difference of 0.013	60	23.4	0.30	5.5
		Rate Difference of 0.02	60	21.8	0.28	7.1
		Rate Difference of 0.03	60	19.9	0.25	9.0
	**Not stratified by time since entering care**					
		Rate Difference of 0.017 ^a^	60	22.2	0.30	6.7
		Gompertz ^a^	60	21.6	0.29	7.3
	**Includes actual Mortality Rates for 60–89**					
		Gompertz	90	21.8	0.90	7.1
		Rate Ratio of 2.0	90	21.5	0.84	7.4
	**Without extrapolation**					
	55+ open-ended Age Group		none	37.2	na	-8.3

^a^ Mortality rates are pooled with data for 1^st^ year of care and 1^st^ year survivors combined

**Fig 7:**
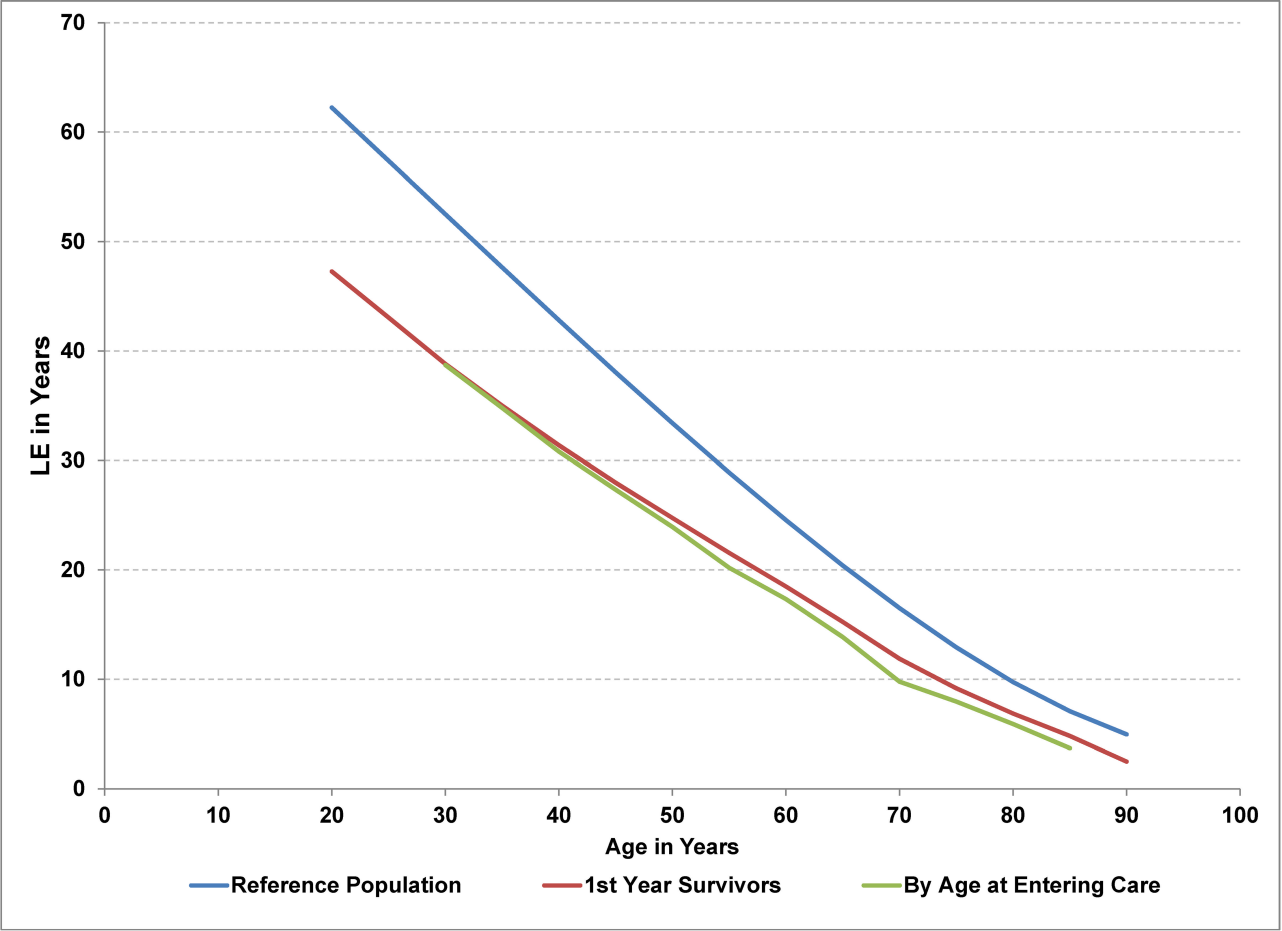
Life expectancy for the HIV cohort versus the general population by age and age at entering care for HIV.

The impact of a higher mortality rate during the first year of care is negligible for younger age groups, although it increases to 2.1 additional YLL for persons entering care at age 70 (Tab G in S1 Tables, Fig 7).

Less than 1% of the HIV cohort entered care before age 20, with the median age at entry closer to 35 years and the average 40 years. The average LE for a person entering HIV care during the 2005 to 2012 period was estimated at 33 years, or equivalently 12 YLL.

The declining trend observed for mortality rates over time translates to increases in LE. The estimated LE for a 20-year old with HIV increased from 16 years based on mortality rates for 1994–95, to 40 years for 2000–2002, 45 for 2006–2007 and 50 years for 2010–2012 (Tab H in S1 Tables, Gompertz projection with extrapolation starting at age 60).

## Interpretation

While there seems to be general agreement that the assumption of constant proportional mortality risks is not a suitable assumption for HIV [6,11,22,23], most LE studies have used either relative survival models with the proportional hazards assumption or an abridged life table approach with the lower limit of the open-ended age group reduced to 55 years [7]. With the expected age at death now exceeding 55 years, there is a need to extrapolate mortality rates to older age groups, and we found that if one assumes that HIV mortality rates are at least as high as those of the general population, uncertainty in the LE estimate due to the choice of extrapolation method was limited to only a few years. Results were also similar when we used the actual age-specific mortality rates for the older age groups with a small population size. The estimated precision of +/- 1 year is reasonable compared to the large bias introduced by using 55+ as the open-ended age group. While the impact of ignoring time since entry (use of pooled mortality rates) resulted in a modest bias of only 1.5 additional years of life lost, this bias could be much larger in other situations where LE is calculated based on CD4 count (due to larger differences in the mortality rate by stratified by time since diagnosis).

### Comparison with other studies

We updated an HIV cohort developed from a linked administrative health database developed by Antoniou and colleagues [4,13]. In addition to confirming Antoniou’s concern about increasing incidence of entering care among persons aged 45 years or older, we found that mortality rates during the first year of care are also considerably elevated for persons entering care at an older age. The elevated mortality hazard during the first year of care for older adults is likely due in part to a lead time or length bias, as the proportion of persons diagnosed asymptomatically would be higher in the younger age groups. A lead time of up to 10 years is possible for regular testers [24]. The shift in the distribution of new cases towards older ages could be due to the higher HIV prevalence and incidence among persons born in the 1960’s (persons born in 1960 turned 45 in 2005). The relatively lower in-care prevalence among persons born after 1970 compared to the 1960’s birth cohorts at comparable ages, suggests that public health interventions have been somewhat effective and that incidence will stabilize at slightly lower rates.

We were able to reproduce the sharp increase in the estimated LE over the early 2000’s observed in other Canadian studies [2,25], but only if we used a 55+ open-ended age group as well. Using the Gompertz projection or other projection options, the estimated increase in LE was considerably moderated. In comparison to Samji’s estimated LE of 51 years for an HIV-positive 20-year old in 2006–2007 [2] our estimate was 45 years in 2006–2007 and 50 years in 2010–2012 (see Tab H in S1 Tables for additional comparisons). Comparison with other studies is limited, as most other studies that used a relative survival model were based on either specialized subpopulations, or cases from high prevalence/low income countries [6,7,26]. A Danish study [5] estimated a LE of 32.5 years for a 25-year old based on mortality rates for the 2000–2005 period, while we obtained a similar estimate of 34.8 years for the same period. A US study [27] that used a similar study design with proportional hazards and stratification by time since diagnosis (less than 1 year versus 1 year or more) estimated LE for persons diagnosed with HIV in 2005 of 22.5 years and 25.5 years for white males. The authors acknowledge that this estimate is low compared to other studies; however, few studies estimate the average LE of a population-based cohort. Using our Ontario HIV cohort for the 2004 to 2006 period, we calculated an average LE of 27 years for newly diagnosed patients, which is comparable to the US results.

Although CD4 count and viral load are strong predictors of mortality risk, studies that looked specifically at the long-term impact of time on cART have not found any evidence of an impact on mortality risks after the initial period of stabilization [11]. An Australian study [22] found that after controlling for current CD4 count, mortality rates did not vary by duration of treatment. A South African study [11] found higher mortality rates during the first three years of cART, while Trickey and colleague [10] found that initial CD4 count was not a significant factor in predicting mortality risks in persons on cART for 10 years or more.

### Limitations

The main limitation was working within the confidentiality constraints of the REB approval that permits ICES to provide external researchers with access to linked health data through their IDAVE system. To avoid residual disclosure, cells sizes of 5 or smaller must be supressed in any published tables. As a result, we chose to provide a minimal dataset for the period of 2005–2012 so that we could publish the age-specific mortality rates used in the calculation of LE. As there were other constraints on the remote access database, namely, limits on potential identifying information such as age at diagnosis, or single year of birth, we requested the age-specific mortality rates for the 2005–2012 period in an auxiliary table (created by ICES staff with direct access to the full linked database). Though use of the remote access database forced us to extrapolate starting at age 60, results were similar to those based on the ICES generated mortality table and the sensitivity analysis of the extrapolation options was richer for this (Table 4). Using 5-year birth cohorts we were able to age the cohort each year by using the mid-year of the range for the representative age. Though the population-years at risk were below the suggested threshold of 5000 for each 5-year age group from 65 to 89 years (Tab F in S1 Tables), the error bar associated with the 95%CI for the LE estimate for age 20 increased only marginally.

As with most disease-specific cohorts, a linked administrative health care database can only identify individuals who are deemed to be in care for the disease. Hence, we were unable to estimate the true incidence and prevalence of HIV infection as diagnosis is often delayed many years. We provided in-care incidence and prevalence numbers by 5-year birth cohorts as supplementary files, as these time-series could be used along with knowledge of the natural history of the disease, asymptomatic testing rates, and back-calculation methods to estimate the incidence and prevalence of infections [28]. Although the Public Health Agency of Canada has estimated that only 80% of persons living with an HIV infection have been diagnosed and only 76% of those diagnosed are on cART, [29,30] one can assume that most of these undiagnosed cases will eventually enter care as the estimates of the median time from sero-conversion to AIDS in the pre-ART period averaged 11 years and for sero-conversion to death 12.5 years [6]. For persons older than age 40, additional screening and timely treatment could eliminate the excess mortality risk observed during the first year of care, potentially saving 1 year of life per person entering care (Tab G in S1 Tables).

We did not have access to clinical records that could be used to determine risk factors for HIV mortality such as disease stage, use of cART, degree of viral suppression, and lifestyle or transmission route that would be available with a clinical cohort. Hence, we have provided the SAS macro in a supplementary file (S1 Zip File) for use by other researchers with access to large clinical cohorts. Though cause of death was not available in the ICES dataset, the ratio of deaths due to HIV/AIDS from the Statistics Canada vital statistics registry to all-cause deaths of persons in care for HIV has declined significantly from 80% in 2000 to 45% in 2012. In another study, the proportion was considerably lower (22% in 2010) among HIV patients on cART for 2 or more years [31]. With the use of a linked database, loss to follow-up was less of a concern than for clinical studies [25] as records were linked with the death file and lost to follow-up rates agreed with interprovincial migration rates [14]. However, there was some uncertainty about the appropriate end date for patients who moved out of the province, as eligibility was only partially maintained.

### Conclusions

In summary, the population-based cohort of persons in care for HIV created from linked administrative health databases and a validated case-finding algorithm provided quality data on trends in the burden of HIV. With deaths among persons treated for many years now mainly due to causes other that AIDS [10] linkage with the death database is required to fully assess mortality risks. The benefits of public health initiatives in screening and prevention are evident in the declining trend in in-care incidence rates for younger adults, though most of this reduction is associated with sustained lower incidence rates in younger adults compared to those born in the 1960s. Health care benefits from improved treatments are also apparent as mortality rates continued to decline despite an aging cohort, though further increases in LE will be moderate as LE approaches that of the general population. Additional screening targeting older adults at risk for HIV transmission could potentially save 1 additional year of life per person entering care after the age of 40 (Tab G in S1 Tables). Use of lower age limits for the open-end age group (such as using a 55+ open-end age group) in an abridged life table calculation of LE significantly overstates LE and is not recommended. Rather, calculation of mortality rates stratified by time since entry (diagnosis or starting cART) and use of mortality rates for older age groups even with a smaller population size or extrapolation of mortality rates to these older age groups is recommended. The Chiang II method easily accommodated input of stratified mortality rates and extrapolation of excess mortality rates.

## Supporting information

S1 Tables Excel workbook with supporting tables and data corresponding to figures. (XLSX)

S1 Zip File SAS macros, sample program and documentation. (ZIP)
