# Supplementary material for: Empirical estimation of life expectancy from a linked health database of adults who entered care for HIV
Source: PLoS One. 2018 Apr 5;13(4):e0195031. doi: 10.1371/journal.pone.0195031 (PMC5886421; doi:10.1371/journal.pone.0195031)
Supplement: S1 Zip File — (ZIP) [file pone.0195031.s002.zip › LifeTable_ChiangII Documentation with references.docx]

Life Expectancy Calculator, SAS Macro %LifeTable_ChiangII

# Purpose:

The purpose of the SAS Macro %LifeTable_ChiangII is to calculate life expectancy (LE) with 95%CI using the Chiang II modification [1-3] and provide built in options to extrapolate mortality rates to older age groups for situations, such as an emerging disease, where the population at older ages is too small to estimate the age-specific mortality rates. The Chiang II method is a modification of Chiang I abridged life table method designed to handle age groups with zero deaths and small populations. This SAS macro is a modification of a SAS macro written by Loukine and colleagues [4] to calculate LE and Health Adjusted Life Expectancy (HALE).

Abridged life table methods require age-specific mortality rates by 5-year age groups. When the mortality rates for the older age groups are not available or the population at risk is small, as is the case for cohorts with chronic medical conditions, extrapolation is recommended [5]. Three extrapolation options are built into the macro: 1) Gompertz’s law (mortality rates increase exponentially with age), 2) extrapolation based on a constant relative mortality rate or rate ratio compared to a reference population, and 3) extrapolation based on a constant excess mortality rate or rate difference compared to the general population.

The macro is designed to calculate LE for disease specific cohorts and will accept mortality rates stratified by time since diagnosis to account for higher mortality rates following diagnosis.

# Instructions to run working example:

# The SAS program HIV_LE_example.sas is included in the zip file to set up the datasets with the age specific mortality data and run the %LifeTable_ChiangII macro. A second macro, %LE1styear, is provided to set up the input (cohort) dataset for stratification by time since diagnosis (entry into care). HIV_LE_example.sas provides a working example with instructions. The user will have to replace the dummy path names for these files with the appropriate path names.

# Data Inputs:

## Reference Population:

The SAS program file Setup_Reference_Mort_Rates.sas provides population and death counts by the standard 5-year age groups (<1yr, 1-4, 5-9, …, 85-89, and 90+) for a total of 20 age groups for males, females and combined. The program creates a SAS DataSet RefMx for input to %LifeTable_ChiangII. The RefMx table can be modified to include additional 5-year age groups (90-94, 95-99, 100+ for example).

## Cohort Population:

The SAS program file Setup_HIV_Mort_Rates.sas provides population and death counts by 5-year age groups stratified by time since diagnosis for our HIV cohort. In this example, male and female were combined and the counts are for a period of 8 years due to small population sizes. The data provided includes 5-year age groups from 20-24 to 85-89 and 90+ though population at risk counts are well below recommended thresholds for many of the older age groups. The two main datasets are: HIVDBT1and HIVDBT0 for 1^st^ year survivors and 1^st^ year of care respectively. HIVDBT1can be input directly to %LifeTable_ChiangII to calculate LE for persons who have survived their 1^st^ year after diagnosis. The LE is calculated for the start of the each age group. Mortality rates for older age groups can be simply ignored (deleted) to start extrapolation at younger age groups. SAS datasets HIVDB55T1 and HIVDB55T0 are created to change the open cohort from 90+ to 55+ for comparison purposes. Again for comparison purposes, the SAS dataset HIVDBallT was created to calculate LE ignoring the stratification by time since diagnosis (byadding the population at risk and deaths counts by age group in the two files (HIVDBT1 and HIVDBT0).

## Cohort Population- stratification by time since diagnosis:

The SAS macro %LE1styear combines the two datasets (HIVDBT1 and HIVDBT0) into one dataset stratified by time since entering care in order to create a single input dataset with age groups and mortality rates stratified by time since diagnosis. Note that %LifeTable_ChiangII must be run once for each age at diagnosis for which LE is to be calculated (for example, the 20-24 year age group is be divided into a 20 and 21-24 year age group for persons developing the disease at age 20 in order to calculate the LE of persons diagnosed at age 20).

Examples are provided in the SAS program file HIV_LE_example.sas.

# Macro Call:

/*

Calculation of LE for persons who:

- have already survived their 1st year of care (input_db=HIVDBT1);
- extrapolation starts at 70 years of age(where=(AgeGroupYr1<70))
- use a constant rate ratio of 2 for the extrapolation (RateRatio=2)
- output LE results to SAS dataset (output_lifetable=LERR2)

Other parameters:

- Reference mortality rates for both sexes combined (input_MxRef= RefMx(where=(sex='T')))
- Age group number 20 is the open ended age group (last_agegroupN=20)
- start with age 20 as the youngest year for which LE is calculated (first_agegroupN=6)
- specify age in years at the start of the age group in order to calculate the number of years in each interval (age_group_yr1 =AgeGroupYr1)
- identify variable names for agegroup, sex, by variables(age_groupYR=AgeGroup, age_groupN=AgeGroupN,sex=Sex, by_variables=,)
- identify the variable names for deaths and population counts (deaths=Deaths1,population=Population1)
- print output (print=Y or print=N)

Other extrapolation options available:

- use a constant excess mortality rate difference of 1% (RateRatio=, RateDiff=0.01, Gompertz=,)
- use the Gompertz law projection (RateRatio=, RateDiff=, Gompertz=Gompertz,)

*/

%***LifeTable_ChiangII***(

input_db=HIVDBT1(where=(AgeGroupYr1<**70**)), input_MxRef=RefMx(where=(sex='T')),

output_lifetable=LERR2,output_desc= %str( Ontario **2005**-**2012**), age_groupYR=AgeGroup, age_groupN=AgeGroupN,sex=Sex,

by_variables=,

deaths=Deaths1,population=Population1,

MxRef=MxRef,

RateRatio=**2**, RateDiff=, Gompertz=,

age_group_yr1=AgeGroupYr1,

first_agegroupN=**6**,

last_agegroupN=**20**,

print=Y);

# Outputs:

| \| \| **Mortality Rates, Log Scale by Age  -   Ontario 2005-2012** \| \| --- \| \| **Input Files: input_db = HIVDBT1(where=(AgeGroupYr1<70)) -  Reference=RefMx(where=(sex='T'))** \| \| **By:  Sex - Extrapolation: RateRatio= 2** \| \| \| --- \| --- \| --- \| --- \| \| \| \| **Sex=T** \| \| --- \| \| \| --- \| --- \| \| \| 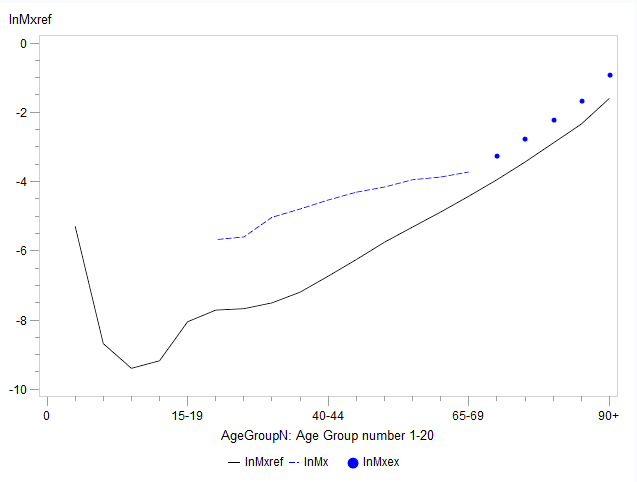 \| \| --- \| \| \| |
| --- | --- | --- | --- | --- | --- | --- | --- | --- | --- |

Figure is plotted on a log scale showing the mortality rate by 5-year age groups for the reference population and the excess mortality associated with the cohort along with the extrapolated values.

| \| \| **Chiang II Abridged Lifetable starting with  Age Group  6** \| \| --- \| \| **Input Files: input_db = HIVDBT1(where=(AgeGroupYr1<70)) -  Reference=RefMx(where=(sex='T'))  -  Ontario 2005-2012** \| \| **By:  Sex - Extrapolation: RateRatio= 2  Outputfile: LERR2** \| \| \| --- \| --- \| --- \| --- \| \| \| \| **Sex=T ExtrapM=RateRatio 2** \| \| --- \| \| \| --- \| --- \| \| \| \| **AgeGroupN** \| **AgeGroupYr1** \| **nMx** \| **Population1** \| **MxRef** \| **RD** \| **RR** \| **ex** \| **LOWER_LE** \| **UPPER_LE** \| **var_ex** \| **Errorbar95CI** \| \| --- \| --- \| --- \| --- \| --- \| --- \| --- \| --- \| --- \| --- \| --- \| --- \| \|  \| 0 \| . \| 1 \| 0.00503 \| . \| . \| . \| . \| . \| . \| . \| \| 1-4 \| 1 \| . \| 1 \| 0.00017 \| . \| . \| . \| . \| . \| . \| . \| \| 5-9 \| 5 \| . \| 1 \| 0.00008 \| . \| . \| . \| . \| . \| . \| . \| \| 10-14 \| 10 \| . \| 1 \| 0.00010 \| . \| . \| . \| . \| . \| . \| . \| \| 15-19 \| 15 \| . \| 1 \| 0.00032 \| . \| . \| . \| . \| . \| . \| . \| \| 20-24 \| 20 \| 0.00342 \| 1,462 \| 0.00045 \| 0.002974 \| 7.6638 \| 47.2766 \| 46.3024 \| 48.2508 \| 0.24704 \| 0.97419 \| \| 25-29 \| 25 \| 0.00369 \| 4,873 \| 0.00046 \| 0.003229 \| 7.9449 \| 43.0489 \| 42.3306 \| 43.7671 \| 0.13430 \| 0.71828 \| \| 30-34 \| 30 \| 0.00651 \| 7,983 \| 0.00055 \| 0.005964 \| 11.8422 \| 38.8047 \| 38.1635 \| 39.4459 \| 0.10702 \| 0.64119 \| \| 35-39 \| 35 \| 0.00827 \| 13,174 \| 0.00075 \| 0.007525 \| 11.0423 \| 35.0067 \| 34.4335 \| 35.5799 \| 0.08552 \| 0.57318 \| \| 40-44 \| 40 \| 0.01073 \| 20,494 \| 0.00119 \| 0.009546 \| 9.0259 \| 31.3799 \| 30.8436 \| 31.9162 \| 0.07487 \| 0.53629 \| \| 45-49 \| 45 \| 0.01345 \| 21,712 \| 0.00192 \| 0.011527 \| 6.9965 \| 27.9728 \| 27.4498 \| 28.4957 \| 0.07119 \| 0.52294 \| \| 50-54 \| 50 \| 0.01561 \| 14,795 \| 0.00317 \| 0.012443 \| 4.9244 \| 24.7453 \| 24.2269 \| 25.2636 \| 0.06993 \| 0.51830 \| \| 55-59 \| 55 \| 0.01929 \| 9,021 \| 0.00492 \| 0.014372 \| 3.9236 \| 21.5524 \| 21.0471 \| 22.0577 \| 0.06647 \| 0.50531 \| \| 60-64 \| 60 \| 0.02085 \| 5,037 \| 0.00758 \| 0.013266 \| 2.7502 \| 18.4829 \| 18.0149 \| 18.9510 \| 0.05703 \| 0.46806 \| \| 65-69 \| 65 \| 0.02412 \| 2,612 \| 0.01198 \| 0.012141 \| 2.0137 \| 15.2404 \| 14.8603 \| 15.6205 \| 0.03761 \| 0.38010 \| \| 70-74 \| 70 \| 0.03852 \| 1 \| 0.01926 \| . \| . \| 11.8755 \| . \| . \| 0.00000 \| . \| \| 75-79 \| 75 \| 0.06425 \| 1 \| 0.03213 \| . \| . \| 8.8737 \| . \| . \| 0.00000 \| . \| \| 80-84 \| 80 \| 0.11109 \| 1 \| 0.05555 \| . \| . \| 6.3133 \| . \| . \| 0.00000 \| . \| \| 85-89 \| 85 \| 0.19225 \| 1 \| 0.09613 \| . \| . \| 4.2459 \| . \| . \| 0.00000 \| . \| \| 90+ \| 90 \| 0.40367 \| 1 \| 0.20184 \| . \| . \| 2.4772 \| . \| . \| . \| . \| \| \| --- \| --- \| --- \| --- \| --- \| --- \| --- \| --- \| --- \| --- \| --- \| --- \| --- \| --- \| --- \| --- \| --- \| --- \| --- \| --- \| --- \| --- \| --- \| --- \| --- \| --- \| --- \| --- \| --- \| --- \| --- \| --- \| --- \| --- \| --- \| --- \| --- \| --- \| --- \| --- \| --- \| --- \| --- \| --- \| --- \| --- \| --- \| --- \| --- \| --- \| --- \| --- \| --- \| --- \| --- \| --- \| --- \| --- \| --- \| --- \| --- \| --- \| --- \| --- \| --- \| --- \| --- \| --- \| --- \| --- \| --- \| --- \| --- \| --- \| --- \| --- \| --- \| --- \| --- \| --- \| --- \| --- \| --- \| --- \| --- \| --- \| --- \| --- \| --- \| --- \| --- \| --- \| --- \| --- \| --- \| --- \| --- \| --- \| --- \| --- \| --- \| --- \| --- \| --- \| --- \| --- \| --- \| --- \| --- \| --- \| --- \| --- \| --- \| --- \| --- \| --- \| --- \| --- \| --- \| --- \| --- \| --- \| --- \| --- \| --- \| --- \| --- \| --- \| --- \| --- \| --- \| --- \| --- \| --- \| --- \| --- \| --- \| --- \| --- \| --- \| --- \| --- \| --- \| --- \| --- \| --- \| --- \| --- \| --- \| --- \| --- \| --- \| --- \| --- \| --- \| --- \| --- \| --- \| --- \| --- \| --- \| --- \| --- \| --- \| --- \| --- \| --- \| --- \| --- \| --- \| --- \| --- \| --- \| --- \| --- \| --- \| --- \| --- \| --- \| --- \| --- \| --- \| --- \| --- \| --- \| --- \| --- \| --- \| --- \| --- \| --- \| --- \| --- \| --- \| --- \| --- \| --- \| --- \| --- \| --- \| --- \| --- \| --- \| --- \| --- \| --- \| --- \| --- \| --- \| --- \| --- \| --- \| --- \| --- \| --- \| --- \| --- \| --- \| --- \| --- \| --- \| --- \| --- \| --- \| --- \| --- \| --- \| --- \| --- \| --- \| --- \| --- \| --- \| --- \| --- \| --- \| --- \| --- \| --- \| --- \| --- \| --- \| --- \| --- \| --- \| --- \| --- \| --- \| --- \| --- \| --- \| --- \| --- \| \| \| |
| --- | --- | --- | --- | --- | --- | --- | --- | --- | --- | --- | --- | --- | --- | --- | --- | --- | --- | --- | --- | --- | --- | --- | --- | --- | --- | --- | --- | --- | --- | --- | --- | --- | --- | --- | --- | --- | --- | --- | --- | --- | --- | --- | --- | --- | --- | --- | --- | --- | --- | --- | --- | --- | --- | --- | --- | --- | --- | --- | --- | --- | --- | --- | --- | --- | --- | --- | --- | --- | --- | --- | --- | --- | --- | --- | --- | --- | --- | --- | --- | --- | --- | --- | --- | --- | --- | --- | --- | --- | --- | --- | --- | --- | --- | --- | --- | --- | --- | --- | --- | --- | --- | --- | --- | --- | --- | --- | --- | --- | --- | --- | --- | --- | --- | --- | --- | --- | --- | --- | --- | --- | --- | --- | --- | --- | --- | --- | --- | --- | --- | --- | --- | --- | --- | --- | --- | --- | --- | --- | --- | --- | --- | --- | --- | --- | --- | --- | --- | --- | --- | --- | --- | --- | --- | --- | --- | --- | --- | --- | --- | --- | --- | --- | --- | --- | --- | --- | --- | --- | --- | --- | --- | --- | --- | --- | --- | --- | --- | --- | --- | --- | --- | --- | --- | --- | --- | --- | --- | --- | --- | --- | --- | --- | --- | --- | --- | --- | --- | --- | --- | --- | --- | --- | --- | --- | --- | --- | --- | --- | --- | --- | --- | --- | --- | --- | --- | --- | --- | --- | --- | --- | --- | --- | --- | --- | --- | --- | --- | --- | --- | --- | --- | --- | --- | --- | --- | --- | --- | --- | --- | --- | --- | --- | --- | --- | --- | --- | --- | --- | --- | --- | --- | --- | --- | --- | --- | --- | --- | --- | --- | --- | --- |

Variable description:

AgeGroupN – Age group number from 1 to 20 is formatted to indicate the age group in years.

AgeGroupYr1 – The starting age in years of the age group

nMx – Age specific Mortality Rate for the cohort with missing values replaced with the extrapolated values for use in calculating LE

Population1 – Age specific population at risk (denominator for mortality rate and used to calculate the variance associated with the LE)

MxRef – Reference Mortality Rate

RD – Calculated Rate Difference

RR – Calculated Rate Ratio

ex – LE, life expectancy

Lower_LE – Lower 95%CI for LE

Upper_LE – Upper 95%CI for LE

Var_ex – Variance of ex (LE). Note that the variance is 0 for the open ended age group and for age groups with extrapolated mortality rates.

ErrorBar95CI – This is the error bar used to plot confidence intervals in EXCEL. We found it convenient to report a LE of 47 +/ 1 year for a 20 year in care for HIV.

Note: The variance estimate for LE does not include the uncertainty associated with the missing mortality rates for older age groups. We recommend using a sensitivity analysis to assess the impact of these missing mortality rates. Please keep in mind that uncertainty associated with mortality rates for younger age groups will have a relatively larger impact on LE estimate. In this example, the population size of the 20-24 age group is small and a significant source of uncertainty.

# References:

1. Eayres D, Williams ES. Evaluation of methodologies for small area life expectancy estimation. Journal of Epidemiology and Community Health. 2004;58: 243-249. doi: 10.1136/jech.2003.009654. Available: <http://jech.bmj.com/content/58/3/243.full>.

2. Toson B, Baker A. Life expectancy at birth: methodological options for small populations. National statistics methodological series. 2003;33. Available: https://www.researchgate.net/profile/Barbara_Toson/publication/241469911_Life_expectancy_at_birth_methodological_options_for_small_populations/links/0c9605334dba390e98000000.pdf.

3. Office of National Statistics, UK. Life Expectancy at Birth and at Age 65 by Local Areas in England and Wales: 2012 to 2014, Trends for England and Wales (National, Regional and Local Areas) in the Average Number of Years People Will Live Beyond their Current Age Measured by "Period Life Expectancy".   . 4 November 2015. Available: https://www.ons.gov.uk/peoplepopulationandcommunity/birthsdeathsandmarriages/lifeexpectancies/bulletins/lifeexpectancyatbirthandatage65bylocalareasinenglandandwales/2015-11-04#references.

4. Loukine L, Waters C, Choi BCK, Ellison J. Impact of diabetes mellitus on life expectancy and health-adjusted life expectancy in Canada. Popul Health Metr. 2012;10. doi: 10.1186/1478-7954-10-7. Available: <http://pophealthmetrics.biomedcentral.com/articles/10.1186/1478-7954-10-7>.

5. Strauss DJ, Vachon PJ, Shavelle RM. Estimation of future mortality rates and life expectancy in chronic medical conditions. J Insur Med. 2005;37: 20-34.
